# Revisiting the Open Sampling format: Improving risky choices through a novel graphical representation

**DOI:** 10.3758/s13423-021-02018-4

**Published:** 2021-11-03

**Authors:** Kevin E. Tiede, Felix Henninger, Pascal J. Kieslich

**Affiliations:** 1 Social Psychology and Decision Sciences, University of Konstanz, P.O. Box 43, 78457 Konstanz, Germany; 2 Graduate School of Decision Sciences, University of Konstanz, Konstanz, Germany; 3 Statistics and Data Science in Social Sciences and the Humanities, Ludwig Maximilian University of Munich, Munich, Germany; 4 Mannheim Centre for European Social Research, University of Mannheim, Mannheim, Germany

**Keywords:** Risky choice, Open Sampling format, Risk communication, Numeracy

## Abstract

When making risky choices, people often fall short of the norm of expected value (EV) maximization. Previous research has shown that presenting options in the Open Sampling (OSa) format, a 10-by-10 matrix of randomly arranged outcomes, can improve choices and reduce decision times. First, the current research aims to replicate and extend the findings on the OSa format. To this end, we compare OSa to the common description-based format as well as further graphical representations, and investigate the resulting accordance with EV maximization and decision time. Second, we study whether people lower (vs. higher) in numeracy, the ability to use probabilistic and mathematical concepts, particularly benefit from a graphical representation of options. We conducted five high-powered studies (total *N* = 1,575) in which participants chose repeatedly between two risky gambles, using different populations and gamble-problem sets. Overall, we could not find a benefit of the OSa format in terms of EV accordance in any of the five studies. However, three studies also tested a novel variant of the OSa format with grouped outcomes and found that it consistently improved EV accordance compared with all other formats. All graphical formats led to faster decisions without harming decision quality. The effects of presentation format were not moderated by numeracy in three of the four studies that assessed numeracy. In conclusion, our research introduces a new presentation format which consistently improves risky choices and can also be used to communicate risks in applied contexts such as medical decision making.

## Introduction

Decisions under risk, where any of several outcomes can occur with known probabilities, are a common occurrence in every-day decision-making, ranging from the decision whether to carry an umbrella (lest it rain), or purchase an insurance (to be safe in an unlikely emergency), to choosing a promising but experimental medical treatment. Despite their frequency and the often substantial consequences involved, systematically suboptimal decisions are a well-documented feature of human risky choices (e.g., Fishburn, 1988; Johnson & Busemeyer, 2010; Newell et al., 2015).

What would an ideal risky choice look like? A common normative benchmark for judging the optimality of a decision is its adherence to maximizing expected value (EV)—that is, choosing the option with the better average outcome in the long run, over an infinite number of independent yet structurally identical repetitions (Baron, 2007). To arrive at this value, every outcome^1^ is weighted with the probability of its occurrence—unlikely outcomes receiving proportionally less weight. These principles derive from only a few simple axioms (Savage, 1954; von Neumann & Morgenstern, 1947), and thus adherence, from a strictly rational perspective, should be uncontroversial.

Descriptively, however, a wealth of evidence documents deviations from this norm (Fishburn, 1988), particularly with regard to the adequate treatment of probabilities. Often, people behave as if they overweighted small probabilities and underweighted medium-to-large probabilities (e.g., Tversky & Kahneman, 1992). Assuming that decision-makers have a finite amount of resources to allocate to any particular task, they might be willing to trade some accuracy if that saves them effort in the decision process (Payne et al., 1993). Thus, reducing effort without compromising decision quality may be a further desirable characteristic pertaining not to the outcome, but to the choice process.

Given the potential weight of risky choices, as well as their omnipresence, the question thus arises whether, and how, they can be improved. This is the domain of risk communication, which assumes that, if communicated correctly, decisions under risk might be better and/or easier. In particular, the presentation format has been investigated as a mechanism to improve choices (for reviews, see Garcia-Retamero & Cokely, 2013, 2017; Spiegelhalter, 2017). If there was a way to represent equivalent information that led to a better understanding and, subsequently, improved decisions, that could improve life outcomes for a wide range of people.

**Fig. 1:**
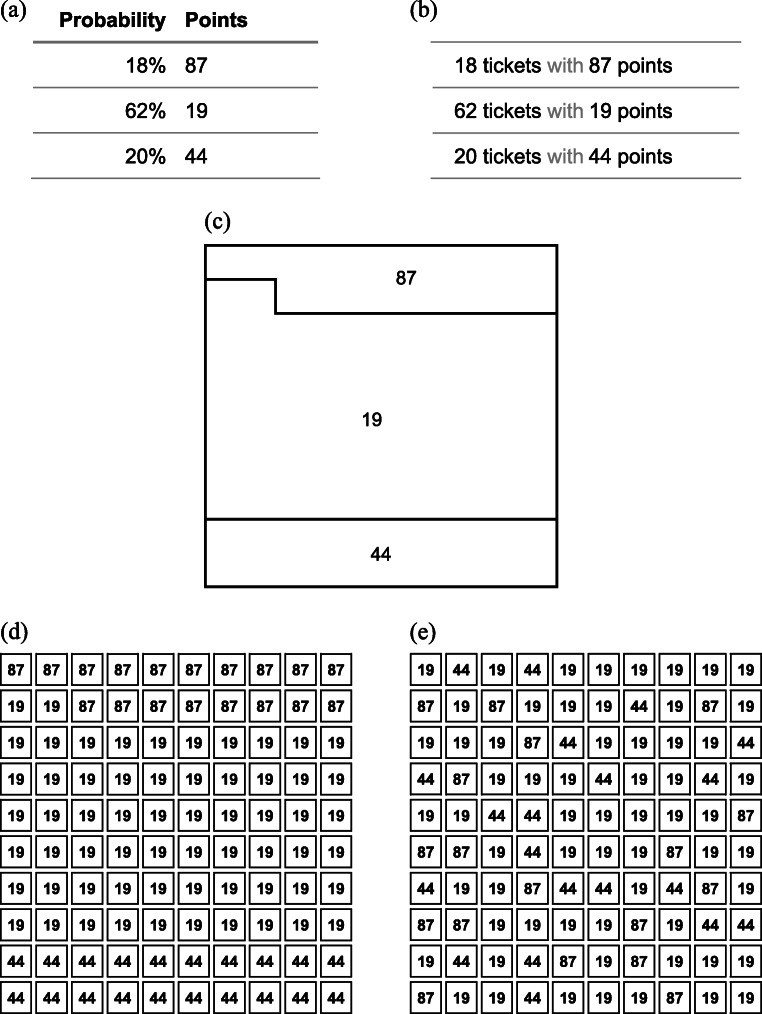
Presentation formats tested in the studies: description (**a**), frequency (**b**), square pie chart (**c**), grouped OSa (**d**), and OSa (**e**). Note that for the description format, in Studies 1a, 1b, and 3 the Points column was presented on the left and the Probability column on the right

In more theoretical terms, if the mode of presentation alone could improve decisions, that would indicate that the issues outlined above may not be inherent flaws in human cognition, but rather the result of an imperfect internal representation of the choice or information sample (Fiedler, 2000; Fiedler & Juslin, 2006). This would also imply that at least part of the observed deviations from the optimal decision might be due to, for example, the typical tabular summary of outcomes next to their probabilities that is commonly used to represent a gamble. Following this argument, Hilbig and Glöckner (2011) introduced the Open Sampling format (OSa; see Fig. 1e), a graphical representation of a lottery as a grid of upturned raffle tickets, each showing its monetary value, with participants instructed that one will be drawn to determine their payoff. In OSa’s 10-by-10 matrix, the frequency of any outcome is proportional to its probability, thus removing the need for listing explicit probabilities (a related format was proposed by Gottlieb et al., 2007, whose “simultaneous” format used a matrix of 20 cells in total instead of 100 in OSa). The randomly shuffled arrangement is designed to facilitate sampling, ensuring that scanning the grid results in (on average) a representative picture of the risky proposition. Hilbig and Glöckner demonstrated in two studies that OSa resulted in the most even treatment of small probabilities compared with a tabular summary (description; see Fig. 1a) and sampling a single outcome at a time (decisions from experience; Hertwig et al., 2004). In both studies, choices “conformed more to the normative hallmark of expected value maximization” (p. 395), in one significantly so, and consistently resulted in the fastest decisions by a substantial margin. This prompted Hilbig and Glöckner to conclude with a joyous “Yes, they can!” (p. 390), an optimistic view of the capabilities of decision-makers confronted with risky choices, constrained by features of the research paradigm rather than their own cognitive capabilities. Starting with the OSa format, our first aim herein is to replicate and extend their results, with the common goal of improving risky choices.

If the advantages of OSa hold widely, the question arises which of its features specifically is responsible for its benefits. While Hilbig and Glöckner motivate their research through a wide range of theoretical considerations, they note that their studies are not conclusive with regard to how OSa achieves its effect, and also call for a closer investigation of the mechanisms at work, stating that “future work will need to trace the main underlying assumptions” (p. 395). Thus, gaining a more detailed picture of the process through which OSa achieves its benefits will provide a deeper understanding of the processes at work, for example whether its reliance on natural frequencies (which have been demonstrated to facilitate Bayesian reasoning; Gigerenzer & Hoffrage, 1995) is the driving factor, or possibly the shuffled arrangement of values (as argued by Hilbig & Glöckner, 2011). Once these processes are known, additional possibilities for improvements may also arise from further, novel, presentation formats. We investigate several such formats in this paper, and test to which degree they improve decisions beyond OSa in terms of EV accordance.

In addition to the external representation, another determinant of decision quality might lie with the individual, in particular their numeracy, the ability to use probabilistic and mathematical concepts (Peters et al., 2006). Numeracy affects life outcomes across a multitude of domains (see Garcia-Retamero et al., 2019; Peters, 2020), among them the degree to which risky choices adhere to the standard of EV maximization (Peters & Bjalkebring, 2015; Traczyk et al., 2018). In contrast to individuals higher in numeracy, those scoring lower struggle with abstract probability information (Peters, 2020). Therefore, they are especially sensitive to how probabilities are presented (e.g., Peters et al., 2006; Peters et al., 2011), and benefit particularly from graphical representations of risks (for a review, see Garcia-Retamero & Cokely, 2017). For instance, Traczyk et al. (2020) found that providing participants with sequentially sampled outcomes improved the accuracy of probability estimates for individuals lower in numeracy (but see Armstrong & Spaniol, 2017). Similarly, the OSa format represents probabilities as the frequency of outcomes in the matrix and may thus allow for easier processing of probabilities compared to explicit numeric values. Because individuals lower in numeracy struggle to process abstract probabilities, one would expect OSa to be especially helpful for them, and numeracy to act as a moderator for the benefits of graphical representations of risky choices.

In the current paper, we revisit the Open Sampling format and reassess its ability to improve choices with regard to the expected value maximization as a benchmark. We also evaluate the effects of additional graphical representations of risky choice, seeking to determine which of OSa’s features may be responsible for the observed benefits. In addition, we test whether the OSa format and other graphical formats are especially beneficial for people lower in numeracy.

## Studies 1a and 1b

In a first set of studies (1a and 1b), which were identical in design but conducted as separate studies, we perform a conceptual replication of the second experiment from Hilbig and Glöckner (2011), with larger samples, and evaluate whether OSa might be especially helpful for individuals lower in numeracy. Thus, the studies compare the typical description-based gamble representation and the OSa format. In line with Hilbig and Glöckner (2011), we expect that accordance with EV maximization is higher and decision times shorter with the OSa format (vs. description). Lastly, we expect that numeracy moderates the effect of format on EV accordance.

### Method

The data for all experiments is openly available at OSF (https://osf.io/2vqnr). All studies have been granted exemption from Institutional Review Board approval by the ethics committee of the University of Konstanz.

#### Participants

For both studies, participants from Germany, mostly undergraduate students, were recruited via mailing lists and social media. In Study 1a, 156 participants completed the experiment. Matching the exclusion criteria of the later studies, we dropped participants who failed one of the two attention checks (i.e., answering more than one of five filler gamble decisions or at least one filler numeracy item incorrectly), responded too quickly to have participated conscientiously (i.e., average decision time <500ms), and/or stated that they did not participate seriously. The final sample consisted of 121 participants (56% female; age: *M* = 29.6 years; range = 18–84 years, *SD* = 13.2). In Study 1b, 301 participants took part. After excluding participants according to the same criteria as above, the final dataset included 272 participants (64% female; age: *M* = 26.1 years; range = 18–70 years, *SD* = 10.8).

#### Procedure

As with the following experiments, we collected data for Studies 1a and 1b online, and participants were requested to complete the study on a computer to ensure a large enough screen size for the OSa format. Following informed consent and demographics, participants were randomly assigned to either the description or OSa format condition. After receiving instructions and making a test decision, participants completed the decision task. Finally, participants filled out the numeracy questionnaire and were asked about the seriousness of their participation. As compensation, participants had the opportunity to enter a lottery in which one participant would be randomly selected. One of this participant’s chosen lotteries would be randomly selected and played out, the participant receiving the outcome as a voucher in Euro.

#### Decision task

In the decision task, participants were asked to imagine drawing a lottery ticket from one of two urns. They were instructed to select the urn they would prefer to draw a ticket from. Participants made 45 choices between two gambles and indicated their choice by pressing a key on the keyboard. Gamble problems were presented either in the description format (see Fig. 1a) or the Open Sampling format (Fig. 1e), depending on the condition.

There were 40 target and five filler gamble problems. Target problems were created randomly following Hilbig and Glöckner (2011, Study 2). In a first step, the number of outcomes for each gamble was randomly chosen between two and five.^2^ Next, those outcomes were associated with values from 0 to 100. Half of the gamble problems included a rare event (i.e., *p* ≤ .05) in one of the two gambles, while the other half of the problems consisted of probabilities larger than .10 only. To avoid the most obvious choices, the ratio of the options’ EVs was restricted to less than or equal to 2. Whereas Hilbig and Glöckner (2011) randomly created the gamble problems on the fly, we created a pool of 4,000 gamble problems, from which we drew 20 rare-outcome pairs and 20 no-rare-outcome pairs for each participant.^3^ In addition, the choice-problem set contained five filler trials, with one superior (EV ratio > 2) and stochastically dominant option. The order of the gamble problems and outcomes within the formats were randomized on a participant and trial level, respectively.

#### Numeracy

We assessed numeracy using the scale developed by Weller et al. (2013). The scale comprised eight numerical tasks^4^ with varying difficulty (e.g., “If the chance of getting a disease is 10%, how many out of 1,000 people would be expected to get the disease?”). In Study 1a, we also included three additional numeracy items with obvious solutions as attention checks. The numeracy score represents the sum of correct answers (excluding the attention check items).

#### Statistical analysis

To test the effect of presentation format and numeracy on EV accordance, we ran linear regression models, with EV accordance (i.e., the proportion of choices in line with EV maximization) as the outcome variable. Presentation format (effect-coded as −.5 = description, .5 = OSa), numeracy (mean-centered), and their interaction were included as predictors. To analyze the effect of presentation format on decision time, we ran the same regression model but with the mean log-transformed response times as the outcome variable. Only nonfiller trials were used for analysis. All findings could be replicated using multilevel models applied to trial-level data.

### Results

Descriptive statistics for all conditions can be found in Table 1. Mean numeracy was 6.2 (*SD* = 1.5) and 6.1 (*SD* = 1.6) in Study 1a and 1b, respectively. Neither study found a significant effect of presentation format on EV accordance (Study 1a: *b* = −0.02, *SE* = 0.02, *p* = .210; Study 1b: *b* = −0.01, *SE* = 0.01, *p* = .201). While numeracy was positively related to EV accordance in both studies (Study 1a: *b* = 0.02, *SE* = 0.01, *p* < .001; Study 1b: *b* = 0.02, *SE* = 0.00, *p* < .001), it moderated the effect of presentation format on EV accordance in Study 1b only (*b* = −0.02, *SE* = 0.01, *p* = .005), and not in Study 1a (*b* = −0.01, *SE* = 0.01, *p* = .380).

**Table 1.** Group sizes, means, and standard deviations (in parentheses) for Studies 1a and 1b

	Description	OSa
Study 1a		
Group size (n)	68	53
EV accordance	.78 (.12)	.77 (.08)
Decision time (s)	14.1 (10.7)	7.3 (6.3)
Study 1b		
Group size (n)	131	141
EV accordance	.77 (.11)	.75 (.08)
Decision time (s)	13.8 (10.6)	10.0 (18.0)

With regard to decision times, participants chose more quickly in the OSa than in the description condition in both studies (Study 1a: *b* = −0.70, *SE* = 0.10, *p* < .001; Study 1b: *b* = −0.59, *SE* = 0.08, *p* < .001).^5^ Individuals higher in numeracy tended to take longer to decide (Study 1a: *b* = 0.06, *SE* = 0.04, *p* = .100; Study 1b: *b* = 0.08, *SE* = 0.03, *p* = .001), but there was no significant interaction of format and numeracy in either study (Study 1a: *b* = −0.08, *SE* = 0.07, *p* = .248; Study 1b: *b* = −0.02, *SE* = 0.05, *p* = .625).

### Discussion

In two studies extending Hilbig and Glöckner (2011), we did not find support for the hypothesis that OSa leads to higher EV accordance, although it generally enabled faster decisions. However, in one of the studies, we found that OSa was particularly helpful for individuals lower in numeracy.

## Studies 2a and 2b

Our failure to replicate the benefit of the Open Sampling format leaves us with the question whether any of the features that make up OSa are advantageous if taken individually, as well as collecting further data to (dis)confirm our null result above.

We therefore conducted Studies 2a and 2b, which vary features of the presentation format systematically, and cover a wider range of possible representations compared with the dichotomy of description and OSa. Specifically, we model the difference between formats as a progression (see Fig. 1), starting from the tabular *description format* (Fig. 1a) and successively adding features. Moving towards OSa, we first replace percentages with frequencies to arrive at a modified description format—if the benefit comes from avoiding probabilities in percent, we would expect this *frequency format* (Fig. 1b) alone to improve choices. Replacing a tabular description with a graphical representation, the *square pie chart format* (Fig. 1c) represents outcomes as sections of a square, with each outcome’s share of the surface corresponding to its probability—if decision-makers are using the relative area as a cue, this representation should be beneficial. The next format, *grouped OSa* (Fig. 1d), subdivides the area into individual outcomes, adopting OSa’s matrix structure—if breaking down probabilities into individual outcomes is effective, we would expect to see improvements at this point. Finally, the *OSa format* (Fig. 1e) shuffles the outcome values, corresponding to the notion of allowing participants to acquire a random, unbiased sample from the distribution of outcomes. Based on the reasoning by Hilbig and Glöckner (2011) that the benefits from OSa stem from its combination of features, we expect an incremental improvement of EV accordance with every added feature. Finally, we hypothesize that graphical formats are particularly beneficial for people lower in numeracy (Garcia-Retamero & Cokely, 2017).

### Method

The methods of Studies 2a and 2b were very similar except for the differences in the sample, the assessment of numeracy, and the language. We follow the preregistration for Study 2b throughout our analysis, which can be found at OSF (https://osf.io/4x5ve).

#### Participants

In Study 2a, 602 German participants took part, recruited online from different websites and social media channels. As preregistered for Study 2b, we excluded participants who chose six or less out of eight filler choices correctly and/or responded too quickly to have participated conscientiously (i.e., average decision time <500ms). Next, we excluded participants who stated that they did not participate seriously, did not understand the task, and/or used a calculator when answering the numeracy questions. Of the 529 remaining participants, 61% were female and on average, they were 32.2 years old (range = 18–81 years, *SD* = 13.0), and 63.7% had at least a bachelor’s degree. For Study 2b, participants residing in the US were recruited via Amazon Mechanical Turk. In total, 512 participants took part in the study. After applying the same exclusion criteria, the data of 410 participants entered analysis. Of these participants, 44% were female and on average, they were 36.9 years old (range = 20–79 years, *SD* = 10.7). In total, 48.3% had a bachelor’s degree or more.

#### Procedure

Following informed consent and demographics, participants were randomly assigned to one of the five format conditions. Then, participants were instructed on the decision task and completed five test decisions. They subsequently made 60 choices in the decision task. In Study 2a, the study was completed at this point. In Study 2b, participants went on to fill out a numeracy questionnaire. At the end of each study, participants were asked about the seriousness of their participation. The compensation differed between studies. In Study 2a, participants’ choices were played out and the payoffs were summed up across the experiment. The ten participants with the highest score received a gift certificate. In Study 2b, one randomly selected choice was played out for each participant and participants received the payoff in $-cents in addition to a fixed $2.50 payment. Study 2a was conducted in German, Study 2b in English.

#### Decision task

The decision task was the same as in Studies 1a and 1b, except for two differences. First, participants were assigned to one of the five format conditions described above (see Fig. 1). Second, the selected gamble problems differed slightly from those in our first set of studies, corresponding to a selection of stimuli more common in studies of risky choice (e.g., Glöckner & Pachur, 2012). Participants were presented with the same set of 60 gamble pairs, with between one and four outcomes and outcome values between 0 and 99 points. The set of gamble problems comprised four subsets taken from different sources. We used 10 two-outcome problems designed to measure risk aversion (Holt & Laury, 2002) and 16 randomly generated two-outcome problems used by Rieskamp (2008). In addition, we randomly generated 26 multiple-outcome gambles with the restriction that one gamble had at least two and the other at least three outcomes. Finally, we included eight filler trials with an obvious superior option (EV ratio >2.0). The order of the gamble problems and outcomes within the formats were randomized on a participant and trial level, respectively.

#### Numeracy

In Study 2b, we assessed numeracy using a 7-item questionnaire, consisting of three items by Schwartz et al. (1997) and a parallel form of the non-adaptive four-item Berlin Numeracy Test (Cokely et al., 2012). This combined measure has shown good discriminability and has been recommended for MTurk samples (Cokely et al., 2012) which we collected data from in this study. The numeracy score represents the sum of correct answers.

#### Statistical analysis

To test the effect of presentation format and numeracy on EV accordance, we again ran a linear regression model with EV accordance as the outcome variable. As preregistered, we included four contrast variables in the regression models to compare the effects of presentation format. The first contrast was designed to capture the difference between all tabular formats (i.e., description and frequency) on one hand, and the graphical formats on the other (i.e., square pie chart, grouped OSa, and original OSa). The second contrast differentiated within the tabular formats, comparing the description to the frequency format. The third contrast compared the square pie chart format with both OSa formats, and the fourth compared grouped OSa and original OSa. Presentation-format contrasts, numeracy (in Study 2b only, mean-centered), and their interactions (in Study 2b only) were included as predictors in the regression model. Decision time was analyzed through the same regression model with mean log-transformed decision times as outcome variable. Only nonfiller trials were used for analysis. As before, we replicated the pattern of results using multilevel models applied to trial-level data.

### Results

**Fig. 2:**
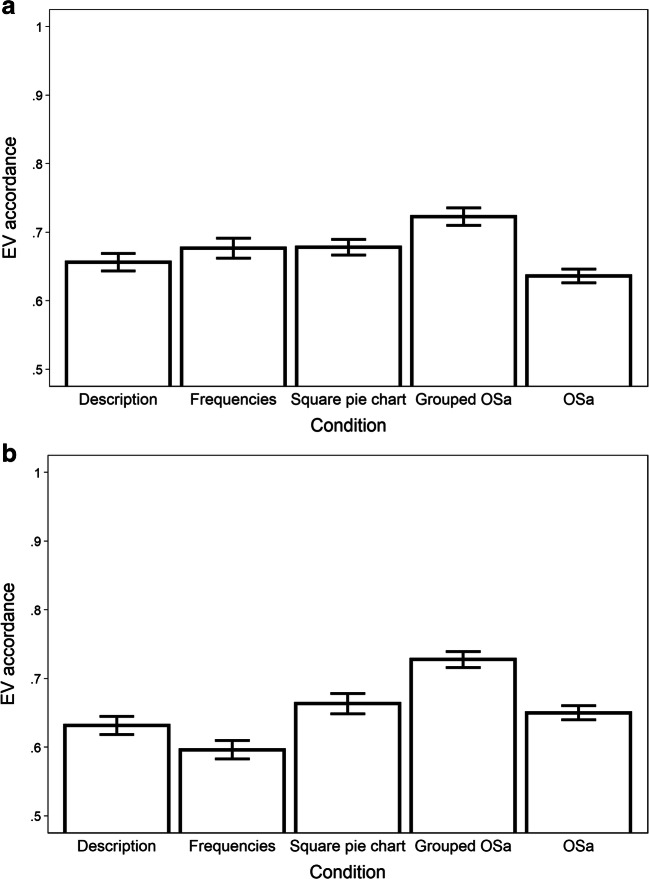
EV accordance results for Study 2a (upper panel) and Study 2b (lower panel; error bars represent one standard error of the mean)

The results are illustrated in Fig. 2, with all descriptive statistics presented in the Appendix Table 5. Results of the regression models can be found in Tables 2 and 3. Regarding EV accordance, the only consistently significant contrast was the one between the grouped and original OSa format, with higher levels of EV accordance in the grouped OSa condition. In Study 2b, graphical formats led to higher EV accordance than tabular formats, but not in Study 2a. As can be seen in Fig. 2, in both studies EV accordance was highest in the grouped OSa condition. The results for numeracy (in Study 2b; *M*_Numeracy_ = 3.5, *SD*_Numeracy_ = 1.6) showed that numeracy was positively associated with EV accordance, but did not significantly interact with any of the format contrasts. Regarding decision times, the three graphical formats led to faster decisions than the two tabular formats, with no other contrasts being significant.^6^ Numeracy was positively related to decision time, but did not interact with any format contrast.

**Table 2:** Regression model results of Study 2a

Predictor	EV accordance			Decision time (log-transformed)		
	*b*	*SE*	*p*	*b*	*SE*	*p*
Intercept	.67	.01	<.001	8.74	0.03	<.001
Tables vs. Graphical formats (Contrast 1)	.00	.00	.273	−0.14	0.01	<.001
Probabilities vs. Frequencies (Contrast 2)	.01	.01	.259	0.06	0.05	.207
Square pie chart vs. OSa formats (Contrast 3)	.00	.00	.923	−0.02	0.02	.324
Grouped OSa vs. OSa (Contrast 4)	−.04	.01	<.001	−0.01	0.04	.769

*Note*. For each contrast, the former group was coded with a negative and the latter with a positive contrast.

**Table 3:** Regression model results of Study 2b

Predictor	EV accordance			Decision time (log-transformed)		
	*b*	*SE*	*p*	*b*	*SE*	*p*
Intercept	.65	.01	<.001	7.90	0.03	<.001
Tables vs. Graphical formats (Contrast 1)	.01	.00	<.001	−0.11	0.01	<.001
Probabilities vs. Frequencies (Contrast 2)	−.02	.01	.073	−0.06	0.04	.132
Square pie chart vs. OSa formats (Contrast 3)	.01	.01	.105	0.00	0.02	.873
Grouped OSa vs. OSa (Contrast 4)	−.04	.01	<.001	−0.03	0.04	.404
Numeracy (Num.)	.01	.00	.036	0.10	0.02	<.001
Contrast 1 × Num.	.00	.00	.942	0.00	0.01	.979
Contrast 2 × Num.	.00	.01	.680	0.02	0.03	.504
Contrast 3 × Num.	.01	.00	.097	0.00	0.02	.781
Contrast 4 × Num.	.00	.01	.870	0.00	0.02	.935

*Note*. For each contrast, the former group was coded with a negative and the latter with a positive contrast.

### Discussion

In sum, we again could not find a benefit of the OSa format in terms of more normative choices. However, to our surprise, the grouped OSa format consistently led to higher EV accordance than any other representation and to shorter decision times than the tabular formats. The results also do not support the hypothesis that the OSa format is particularly beneficial for people lower in numeracy.

## Study 3

Following our results above, and the unexpected benefit of the grouped OSa format in particular, we endeavored to replicate our results once more in a fully preregistered study with gambles that follow Hilbig and Glöckner’s approach (2011, Study 2), focusing on the difference between the description format, OSa, and our newly identified contender. Based on our empirical results, we now expect the grouped OSa format to improve EV accordance compared to the description and the OSa format, and test whether the grouped OSa format benefits less numerate individuals in particular.

### Method

#### Sample, procedure, decision task, and numeracy assessment

The preregistration for Study 3 can be found at OSF (https://osf.io/5rfuj). For Study 3, 285 participants from Germany completed the experiment, recruited via mailing lists and social media, 243 of which remained in the dataset after applying the same exclusion criteria as in Studies 1a and 1b. Most participants were undergraduate students, 72% were women and participants were on average 28.2 years old (range = 18–88 years, *SD* = 12.5). The procedure, decision task, gamble pairs, numeracy scale, participant recruitment, and incentivization scheme were the same as for Study 1a, except for the addition of a grouped OSa condition, which was identical to Studies 2a and 2b.

#### Statistical analysis

To test the effect of presentation format and numeracy on EV accordance and decision time, we conducted the same analyses as for Study 2b, adapting contrasts to account for the reduced number of conditions. Specifically, the first contrast compared the description format with the two OSa formats, whereas the second compared the grouped OSa format and the original OSa format. Presentation-format contrasts, numeracy (mean-centered), and their interactions were included as predictors in the regression models. Only nonfiller trials were used for analysis. When we analyzed the trial-level data using multilevel models, the pattern of results remained.

### Results

**Fig. 3:**
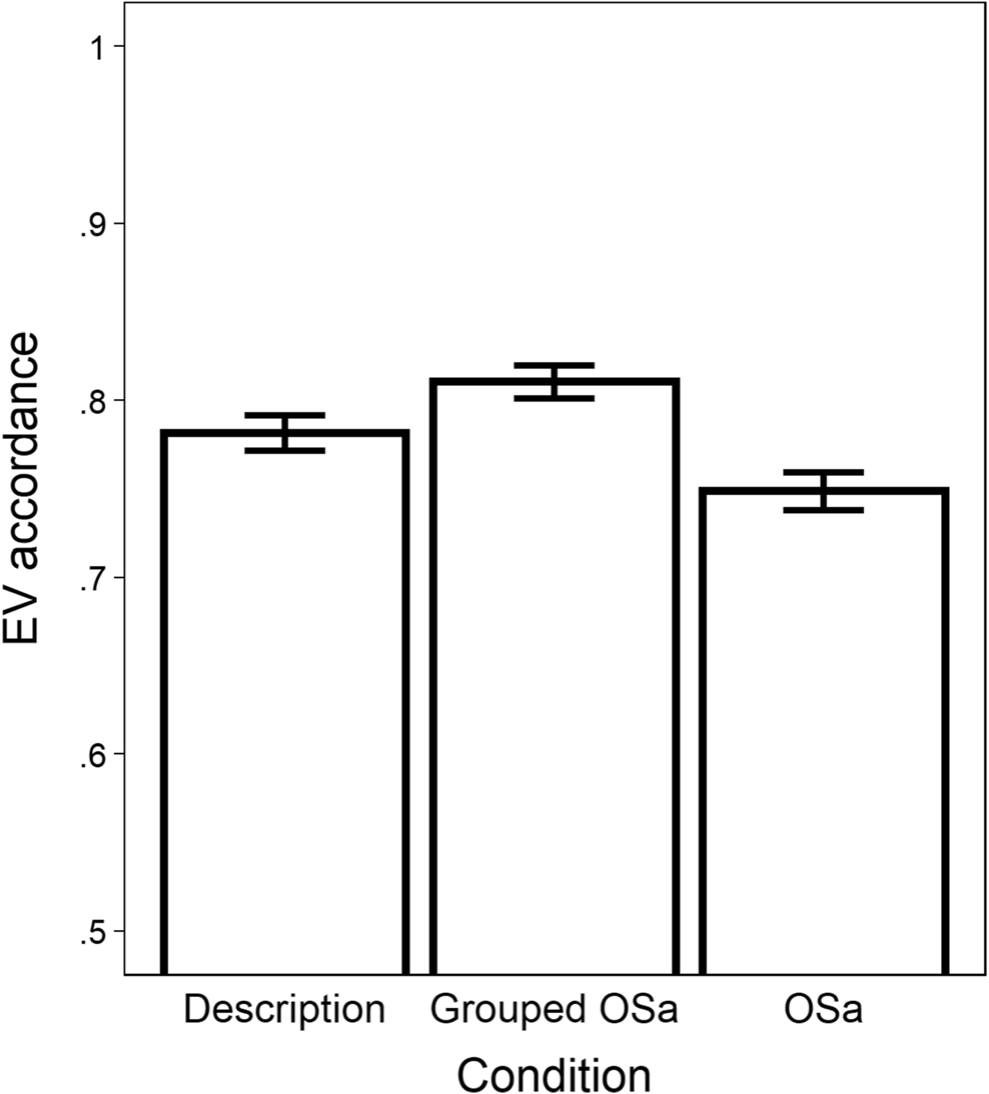
EV accordance results for Study 3 (error bars represent one standard error of the mean)

The results are illustrated in Fig. 3 (see Appendix for descriptive values). Results of the regression model can be found in Table 4. Mean numeracy was 6.2 (*SD* = 1.6). People did not make more normative choices with OSa formats in aggregate, compared to the description format. However, EV accordance was higher with the grouped OSa format than with the original OSa format. Numeracy was positively associated with EV accordance, but did not moderate the effect of presentation format on EV accordance.

**Table 4:** Regression model results of Study 3

Predictor	EV accordance			Decision time (log-transformed)		
	*b*	*SE*	*p*	*b*	*SE*	*p*
Intercept	.79	.01	<.001	8.71	0.04	<.001
Probabilities vs. OSa formats (Contrast 1)	.00	.00	.980	−0.20	0.03	<.001
Grouped OSa vs. OSa (Contrast 2)	−.03	.01	<.001	−0.06	0.05	.200
Numeracy (Num.)	.01	.00	<.001	0.01	0.02	.595
Contrast 1 × Num.	.00	.00	.484	0.01	0.02	.465
Contrast 2 × Num.	.00	.00	.362	−0.03	0.03	.291

*Note*. For each contrast, the former group was coded with a negative contrast and the latter with a positive contrast.

**Table 5:** Group size, EV accordance, and decision time for all conditions of Studies 2a, 2b, and 3

	Description	Frequencies	Square pie chart	Grouped OSa	OSa
Study 2a					
Group size (n)	104	84	114	99	128
EV accordance	.66 (.13)	.68 (.14)	.68 (.12)	.72 (.13)	.64 (.11)
Decision time (s)	13.7 (12.9)	25.3 (100.4)	7.8 (7.8)	7.6 (9.1)	9.4 (20.4)
Study 2b					
Group size (n)	77	84	69	93	87
EV accordance	.63 (.12)	.60 (.12)	.66 (.12)	.73 (.11)	.65 (.10)
Decision time (s)	5.2 (2.9)	4.8 (3.1)	2.8 (1.6)	2.9 (1.6)	3.0 (2.2)
Study 3					
Group size (n)	79	–	–	84	80
EV accordance	.79 (.09)	–	–	.82 (.09)	.76 (.09)
Decision time (s)	12.6 (7.7)	–	–	8.5 (10.9)	7.2 (7.1)

*Note.* For EV accordance and decision time, means are presented, with standard deviations in parentheses.

The OSa formats led to shorter decision times than the description format^7^, whereas there was no difference between the OSa formats. Lastly, the main effect of numeracy on decision time and its interaction effects with format were not significant.

### Discussion

In a fully preregistered study, we replicated our earlier finding that grouping outcomes in the OSa matrix, rather than distributing them randomly, led to higher EV accordance. Both grouped OSa and OSa led to faster choices without harming decision quality. Again, we found no moderating influence of numeracy.

## General discussion

The format in which a risky decision is presented can change, and potentially improve, the resulting choice. We evaluated the Open Sampling format (Hilbig & Glöckner, 2011), a graphical representation of lotteries, in terms of its ability to improve EV accordance. Across five high-powered experiments with different samples and gamble sets, we found no support for a general improvement in decisions in the original OSa format with randomly arranged outcome values compared with a tabular summary, even for individuals lower in numeracy. This runs counter to the original claim of a general benefit of OSa, which we could not replicate even with structurally identical materials. However, when investigating a wide range of representations of risky choice comprising different features present in OSa, we unexpectedly identified a grouped variant of OSa that consistently improved choices compared with both the original OSa format and a tabular description. Finally, we found that graphical formats consistently led to faster choices.

The lack of support for an advantage of OSa, combined with the benefits for the grouped variant, raises the question whether random sampling from visible outcomes can improve choices alone. Indeed, benefits of grouping have been demonstrated for dichotomous icon arrays (Ancker et al., 2011; Wright et al., 2009), an effect we herein extend to continuous outcomes. Our pattern of results appears to imply that decision-makers are able to construct a cognitive representation of a risky decision without a format that, by preshuffling outcomes, facilitates drawing “large and representative samples” (Hilbig & Glöckner, 2011, p. 391; see Fiedler, 2000). That, on the other hand, we could not observe similar benefits from representing probabilities as frequencies or as areas is consistent with previous findings (e.g., Birnbaum, 2004; Camilleri & Newell, 2011).

A very clear pattern in our data is the consistent finding that all graphical formats result in faster decisions compared with tabular representations.^8^ This pattern of decision times is in line with the notion that OSa formats enable fast scanning of the matrix (Hilbig & Glöckner, 2011). Combined with the fact that no graphical representation fared worse than a table with regard to EV accordance, one could argue that graphical formats are at least more efficient conveyors of information. Given the self-paced nature of our tasks, one might further speculate that decision-makers parlayed that efficiency into increased speed rather than instead taking a comparable amount of time as did those in the tabular conditions, and improving choices beyond their level.

While we found a consistent positive association of numeracy and EV accordance, numeracy did not reliably moderate the effect of format on EV accordance. This finding is in contrast to previous findings that less (vs. more) numerate people are generally more sensitive to the way information is presented (Peters et al., 2011; Peters et al., 2006; but there have been mixed results in decisions from experience, see Armstrong & Spaniol, 2017; Traczyk et al., 2020). Although numeracy values were relatively high and thus negatively skewed in three of our four studies (which might have reduced the chance of uncovering a moderation effect), there was no evidence for the moderation in a somewhat less numerate MTurk sample either. Given a consistent benefit of the grouped OSa format, our results also point to a potential strength of this representation in that it helps people of all levels of numeracy make better decisions.

In our study, we focused on improving EV accordance, which is frequently considered a normative benchmark of rational choice. However, we acknowledge that decision-makers might pursue different goals. Therefore, other formats (including the original OSa format) might benefit criteria which we have not taken into account. Future research is needed to understand how the original and the grouped OSa format affect the use of choice strategies and other decision outcomes.

Given that the grouped OSa format consistently improved choices both in terms of EV accordance and decision time, our research not only contributes to the understanding of the effects of presentation formats on choice. It also introduces a new format which can be used to convey risks in applied fields such as in risk communication and thus may even help to improve people’s decisions in everyday life.

## Footnotes

1 In our paper, we deal with lotteries and translate the numerical magnitude of the outcome directly into a measure of utility. Modern models of risky choice assume that marginal utility reduces with the value of an outcome, and that the relationship is different for gains and losses (Tversky & Kahneman, 1992), but for gains in a moderate range, the assumption of a linear relationship is common (Birnbaum, 1999, 2004).

2 Hilbig and Glöckner (2011) used a range of one to five outcomes.

3 To ensure the difficulty of the choices was comparable between participants, lottery pairs were sorted by their EV ratio, creating bins of each 100 lottery pairs with similar EV ratios. For each participant, one lottery pair from each bin was randomly selected thus covering the whole range of EV ratios for each participant.

4 The scale by Weller et al. (2013) contains two of the three items of the Cognitive Reflection Test (CRT; Frederick, 2005). We also included the third item but did not analyze it.

5 Exploratory analyses revealed that this difference was significant from the first trial onward and increased throughout the experiment (for details, see the supplementary analyses provided in the OSF repository).

6 As in the previous studies, the difference was significant in the first trial and increased throughout the experiment (for details, see supplementary analyses).

7 Again, this difference was significant in the first trial and increased throughout the experiment (for details, see supplementary analyses).

8 Interestingly, this is in contrast to Garcia-Retamero et al. (2016), who found that graphical formats led to increased deliberation time, but their tasks and presentation format differed from our study, and the sample consisted exclusively of medical professionals.

## Declarations

### ^1^Ethics approval

All studies have been granted exemption from IRB approval by the ethics committee of the University of Konstanz (RefNo: IRB20KN012-03/w) and were performed in line with the principles of the Declaration of Helsinki.
